# From AI labels to trust: cognitive pathways linking authenticity judgments, risk perception, and trust in AI-generated videos

**DOI:** 10.3389/fpsyg.2026.1975796

**Published:** 2026-10-20

**Authors:** Jiabin Fu, Yi Zhang, Shu Zhang

**Affiliations:** College of Communication, Weifang University, Weifang, Shandong, China

**Keywords:** AI-generated content, artificial intelligence disclosure, cognitive processing, perceived authenticity, perceived risk, transparency labels, trust

## Abstract

**Background:**

The rapid advancement of artificial intelligence-generated content (AIGC) has raised concerns regarding the authenticity and trustworthiness of synthetic media. Although AI disclosure labels are increasingly used as transparency mechanisms, the cognitive processes through which these cues influence trust formation remain unclear.

**Objective:**

This study aimed to develop and test a cognitive pathway model explaining how AI disclosure labels, authenticity judgments, perceived risk, cognitive processing styles, and literacy factors shape trust toward AI-generated videos.

**Methods:**

A 2 × 2 between-subjects experiment was conducted with 617 participants randomly assigned to four conditions involving AI-generated instructional videos with different AI label and video quality combinations. Participants evaluated authenticity, systematic and heuristic processing, trust, perceived risk, adoption intention, sharing intention, AI literacy, and media literacy. Regression, bootstrapped mediation, and moderation analyses were performed.

**Results:**

AI disclosure labels significantly reduced perceived authenticity but did not directly affect trust, adoption, sharing intention, or perceived risk. Trust formation occurred through cognitive pathways: systematic processing increased trust, whereas heuristic processing reduced trust. Authenticity enhanced trust and lowered perceived risk, with perceived risk mediating the authenticity–trust relationship. Trust positively predicted adoption and sharing intentions, while AI and media literacy moderated selected relationships.

**Conclusion:**

Trust in AI-generated videos was not directly determined by transparency labels alone. Instead, the observed associations among authenticity judgments, perceived risk, information processing, and trust were consistent with a multi-stage cognitive interpretation, although the cross-sectional post-exposure measurement of these constructs does not establish their temporal or causal sequence.

## 1 Introduction

The rapid advancement of artificial intelligence (AI) generative technologies has fundamentally changed the production, distribution, and consumption of digital media (Hancock et al., 2020). Recent generative AI systems are capable of producing highly realistic videos (Westerlund, 2019) that closely resemble human-created audiovisual content, creating new opportunities in education, communication, entertainment, and information sharing. However, the increasing realism of AI-generated media also raises important concerns regarding authenticity assessment (Vaccari and Chadwick, 2020), credibility evaluation, and user trust.

As synthetic content becomes more difficult to distinguish from human-produced material, understanding how individuals interpret and evaluate AI-generated videos has become a critical research priority. Trust represents a central challenge (Glikson and Woolley, 2020) in the adoption and responsible use of AI-generated media. Unlike traditional digital content, AI-generated videos introduce uncertainty regarding source origin, production process, and informational reliability (Fogg and Tseng, 1999). Users must evaluate not only the content itself but also whether the content should be considered authentic, trustworthy, and safe for further engagement.

Previous research indicates that users rely substantially on audiovisual and perceptual cues (Xu and Sundar, 2016) when judging whether synthetic media is authentic or credible. Experimental studies of deepfake and AI-generated video have shown that video characteristics such as visual quality, realism, editing, and audiovisual features can influence credibility and authenticity judgments, while increasingly realistic synthetic content can also make discrimination between authentic and artificial media more difficult. Accordingly, perceptual realism may contribute to credibility judgments without necessarily providing reliable evidence that the underlying content is authentic.

Processing fluency provides an additional theoretical basis for considering video quality in judgments of AI-generated content.

Processing fluency refers to the subjective ease (Petty and Cacioppo, 1986) with which information is perceived and cognitively processed. Research has shown that information that is easier to process may be judged as more familiar, credible, or true, even when processing ease itself is not diagnostic of factual accuracy. Cognitive fluency can contribute to truth judgments and related truthiness effects, while broader research indicates that fluency may function as a metacognitive cue (Sundar, 2008) during credibility evaluation. In audiovisual environments, presentation characteristics such as visual clarity, sharpness, and smoothness may therefore alter processing ease and, in turn, users’ evaluations of the content. From this perspective, higher video quality could potentially enhance perceived authenticity or credibility by making AI-generated content easier to process, whereas degraded presentation quality could increase processing difficulty. Accordingly, the manipulation of video quality in the present experiment was intended not only as a test of perceptual realism but also as a potential source of processing fluency during credibility evaluation.

One increasingly discussed approach to addressing uncertainty surrounding AI-generated content is the implementation of transparency mechanisms (Morley et al., 2020), particularly AI disclosure labels. Disclosure labels provide users with information regarding the origin of digital content and are intended to support informed evaluation and responsible use. However, research on AI disclosure has produced mixed findings, suggesting that transparency cues may generate both potential benefits and costs depending on how users interpret information about AI involvement. On one hand, disclosure labels indicating that content was generated or assisted by AI may reduce perceived authenticity because users may associate artificial production with reduced human involvement, originality, or accountability.

From this perspective, AI labels function as provenance cues that shift attention from the content itself toward the production process (Diakopoulos, 2016) and may increase concerns regarding reliability or manipulation. On the other hand, transparency does not necessarily reduce trust. Research on trustworthy artificial intelligence suggests that transparency mechanisms can support informed evaluation, appropriate reliance (Lee and See, 2004), and calibrated trust (Hoff and Bashir, 2015) when they provide meaningful information (Rai, 2020) about system involvement, limitations, and accountability.

Rather than uniformly increasing or decreasing trust, disclosure information may help users adjust confidence according to available evidence and contextual cues. Recent research further indicates that AI disclosure effects depend on contextual factors (Kizilcec, 2016), including disclosure wording, placement, whether AI is presented as assistance or replacement, the type of content being evaluated, and users’ prior beliefs or familiarity with AI technologies (Liao et al., 2020). Therefore, AI disclosure should not be considered an inherently positive or negative trust cue; instead, its effects may emerge through users’ interpretation of authenticity, risk, and credibility-related information.

Existing studies have primarily examined whether AI-generated content is accepted, rejected, or accurately identified as artificial. Less is known about the cognitive mechanisms (Shin, 2021) through which disclosure information influences trust judgments. In particular, it remains unclear how external signals, such as AI labels and media quality, interact with internal cognitive processes (Nass and Moon, 2000), including systematic evaluation, heuristic judgment, perceived risk assessment, and authenticity perception. Addressing this gap is important because effective AI transparency strategies require more than simply informing users that content is AI-generated; they must also consider how users cognitively process and interpret such information.

The Heuristic–Systematic Model (HSM) provides a useful theoretical framework for explaining how individuals evaluate AI-generated content. According to HSM, individuals process information through two complementary pathways. Systematic processing involves analytical, effortful evaluation of information, whereas heuristic processing relies on simplified cues (Reeves and Reeves, 1996) and intuitive judgments. In AI-generated media environments, users may initially rely on visible cues such as disclosure labels and video quality, but subsequent cognitive processing may determine whether they develop trust or skepticism toward the content. Video quality may also alter the subjective ease with which audiovisual information is processed.

Such fluency can itself operate as a heuristic cue, potentially affecting credibility judgments without requiring extensive systematic analysis. Cognitive load may further shape the way individuals process AI-generated information. In the present study, cognitive load refers to the perceived mental effort required to understand and evaluate the video content. When evaluation requires greater mental effort, individuals may have fewer cognitive resources available for detailed systematic processing and may rely more strongly on simplified heuristic cues. Conversely, lower cognitive load may facilitate more deliberate evaluation of information.

Because cognitive load was not specified as a primary outcome of the experimental manipulation, it was included as a supplementary cognitive-processing variable to clarify how authenticity judgments may relate to systematic and heuristic processing. Accordingly, relationships involving cognitive load were examined as supplementary analyses rather than as formal hypothesis tests. Perceived risk was included because trust judgments toward AI-generated content are likely to involve not only assessments of authenticity but also evaluations of potential uncertainty, misinformation, manipulation, or inappropriate reliance.

In this context, two videos may appear similarly authentic while differing in the degree of risk users associate with relying on or disseminating their content. Perceived risk therefore provides a theoretically distinct mechanism (McKnight et al., 2002) linking authenticity judgments with trust, allowing the model to capture users’ evaluation of possible negative consequences in addition to their assessment of whether the content appears genuine. Perceived authenticity is another important psychological factor influencing trust in synthetic media. When users perceive AI-generated content as authentic and reliable, uncertainty and perceived risk may decrease, leading to greater trust. Conversely, reduced authenticity perceptions may increase concerns regarding manipulation and reliability (Chesney and Citron, 1820), thereby decreasing trust.

Although previous studies have investigated authenticity judgments and AI transparency separately, limited research has examined authenticity as a mechanism connecting AI disclosure, cognitive processing, perceived risk, and trust. To address these gaps, the present study develops and tests a cognitive pathway model explaining how users evaluate AI-generated videos. Specifically, we conducted a 2 × 2 between-subjects experiment manipulating AI disclosure labels (present vs. absent) and video quality (high vs. low). Participants evaluated AI-generated instructional videos and reported their perceptions of authenticity, cognitive processing strategies, trust, perceived risk, adoption intention, and sharing intention.

The study further examines how perceived authenticity, systematic and heuristic processing, perceived risk, and trust are related within the proposed cognitive pathway framework. Cognitive load is additionally examined as a supplementary processing variable that may help explain variation in systematic and heuristic evaluation. Behavioral responses were examined through both adoption intention and sharing intention because these outcomes represent conceptually distinct forms of engagement with AI-generated content. Adoption intention reflects willingness to use or rely on the information personally, whereas sharing intention reflects willingness to transmit the content to others.

This distinction is particularly relevant for synthetic media because content may be accepted for personal use without necessarily being considered appropriate for further dissemination. AI literacy and media literacy were included as potential boundary conditions because users differ in their ability to understand how AI-generated content is produced and to critically evaluate mediated information. Individuals with greater AI literacy may interpret disclosure labels differently because they possess more knowledge about generative-AI systems, while greater media literacy may support more critical evaluation of credibility, source cues, and potential manipulation. Examining these literacy variables therefore allows the study to test whether responses to AI disclosure depend partly on users’ pre-existing evaluative competencies rather than assuming that disclosure operates uniformly across all individuals.

This study makes several theoretical and practical contributions. First, it extends AI transparency research by demonstrating that disclosure cues may influence early authenticity judgments without directly determining trust. Second, it integrates cognitive processing theory and risk perception perspectives to explain how trust develops during evaluation of AI-generated media. Third, it provides practical implications for designing effective AI disclosure strategies by showing that transparency mechanisms should consider users’ cognitive interpretation processes rather than relying solely on the presence of labels.

Based on this theoretical framework, we propose the following hypotheses:

*H1*: AI-generated content labels negatively influence users’ perceived authenticity of AI-generated videos.

*H2*: Perceived authenticity is positively associated with trust and negatively associated with perceived risk.

*H3*: Systematic processing is positively associated with trust, whereas heuristic processing is negatively associated with trust.

*H4*: Perceived risk mediates the relationship between perceived authenticity and trust.

*H5*: Trust positively predicts users’ intention to adopt AI-generated videos.

*H6*: Trust positively predicts users’ intention to share AI-generated videos.

*H7*: AI literacy and media literacy moderate selected relationships between AI disclosure labels and users’ trust and behavioral responses.

Figure 1 summarizes the proposed conceptual framework by linking the experimental cues to perceived authenticity, cognitive processing, perceived risk, trust, and downstream behavioral intentions, while depicting AI literacy and media literacy as moderators of selected relationships.

**Figure 1:**
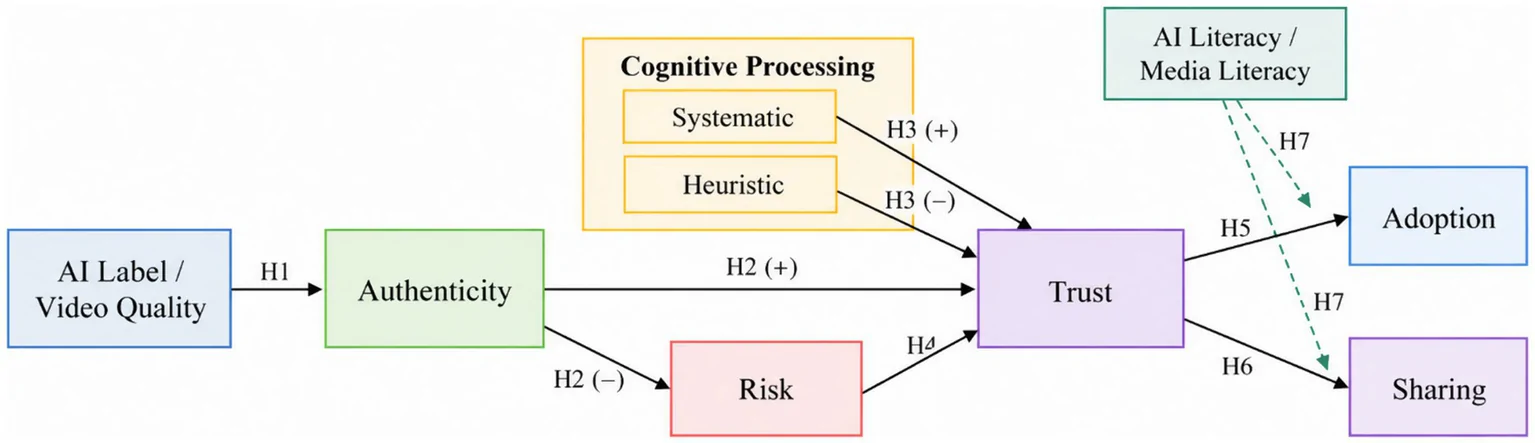
Conceptual model of cognitive pathways linking AI disclosure, authenticity judgments, cognitive processing, perceived risk, trust, and behavioral intentions. Solid arrows represent hypothesized direct or mediated relationships, whereas dashed arrows represent moderation by AI literacy and media literacy.

## 2 Methods

### 2.1 Study framework and experimental resources

This study employed a 2 × 2 between-subjects experimental design manipulating AI disclosure label presence (present vs. absent) and video quality (high vs. low). Participants were randomly assigned to one of four conditions: low quality without an AI label, low quality with an AI label, high quality without an AI label, or high quality with an AI label. The experimental stimulus was an AI-generated instructional video explaining the Pomodoro method. A common script and narrative sequence were prepared by the research team and used to generate the base stimulus with OpenAI Sora.

The same informational content, scene order, captions, narration, and duration were retained across all four experimental conditions. The final video duration was 5 min and 24 s (05:24). After generation, the base video was reviewed and standardized using Adobe Premiere Pro (Version 2024). Post-production involved trimming, caption placement, audio synchronization, normalization, resizing, and standard compression. No substantive changes were made to the instructional content across experimental conditions. Video quality was manipulated from the same standardized source file. The high-quality version was exported at 1080p resolution (1,920 × 1,080 pixels), 30 fps, and H.264 codec at a high bitrate of 12 Mbps, whereas the low-quality version was exported at 360p resolution (640 × 360 pixels), 24 fps, and H.264 codec at a heavily compressed bitrate of 800 kbps with added mild downscaling blur. The manipulation affected resolution, compression artifacts, overall sharpness, and visual clarity. Audio characteristics were identical across the two quality conditions, including a 44.1 kHz sampling rate, 192 kbps stereo bitrate, and standard AAC channel configuration.

Visual content, scene sequence, captions, narration, duration, and instructional complexity were held constant. Thus, the intended difference between the high- and low-quality conditions concerned presentation quality rather than informational content. AI disclosure was manipulated independently of video quality. In the label-present condition, the exact wording “AI-Generated Content/Synthetic Video” appeared in the upper-left corner of the video frame. The label was displayed using Arial font family, 16 px approximate font height, white text color, and a black background with 70% opacity, providing high contrast (white text on a dark opaque background). The label was visible throughout the entire video. The label occupied approximately 5% of the total frame area and did not obscure instructional content.

In the label-absent condition, no disclosure label was displayed. All other video characteristics were identical within each quality condition. Following random assignment, participants viewed the instructional video corresponding to their experimental condition and then completed a questionnaire assessing perceived authenticity, cognitive load, systematic processing, heuristic processing, trust, perceived risk, adoption intention, sharing intention, AI literacy, and media literacy. The video-quality manipulation was theoretically motivated by research on processing fluency, which suggests that perceptually easier-to-process information may influence credibility and truth judgments. By varying visual clarity while holding informational content constant, the experiment therefore allowed us to examine whether presentation quality functioned as a perceptual cue associated with subsequent authenticity, trust, and cognitive-processing judgments.

Figure 2 illustrates the four experimental conditions created by crossing video quality (low vs. high) with AI disclosure label presence (absent vs. present). The schematic shows the approximate participant view, including the position and wording of the AI disclosure label and the relative visual appearance of the high- and low-quality video conditions. The figure is intended to aid replicability and does not reproduce the full original stimulus frame-by-frame. This figure therefore documents the stimulus structure and label placement used to implement the factorial manipulation and supports reproducibility of the experimental setup.

**Figure 2:**
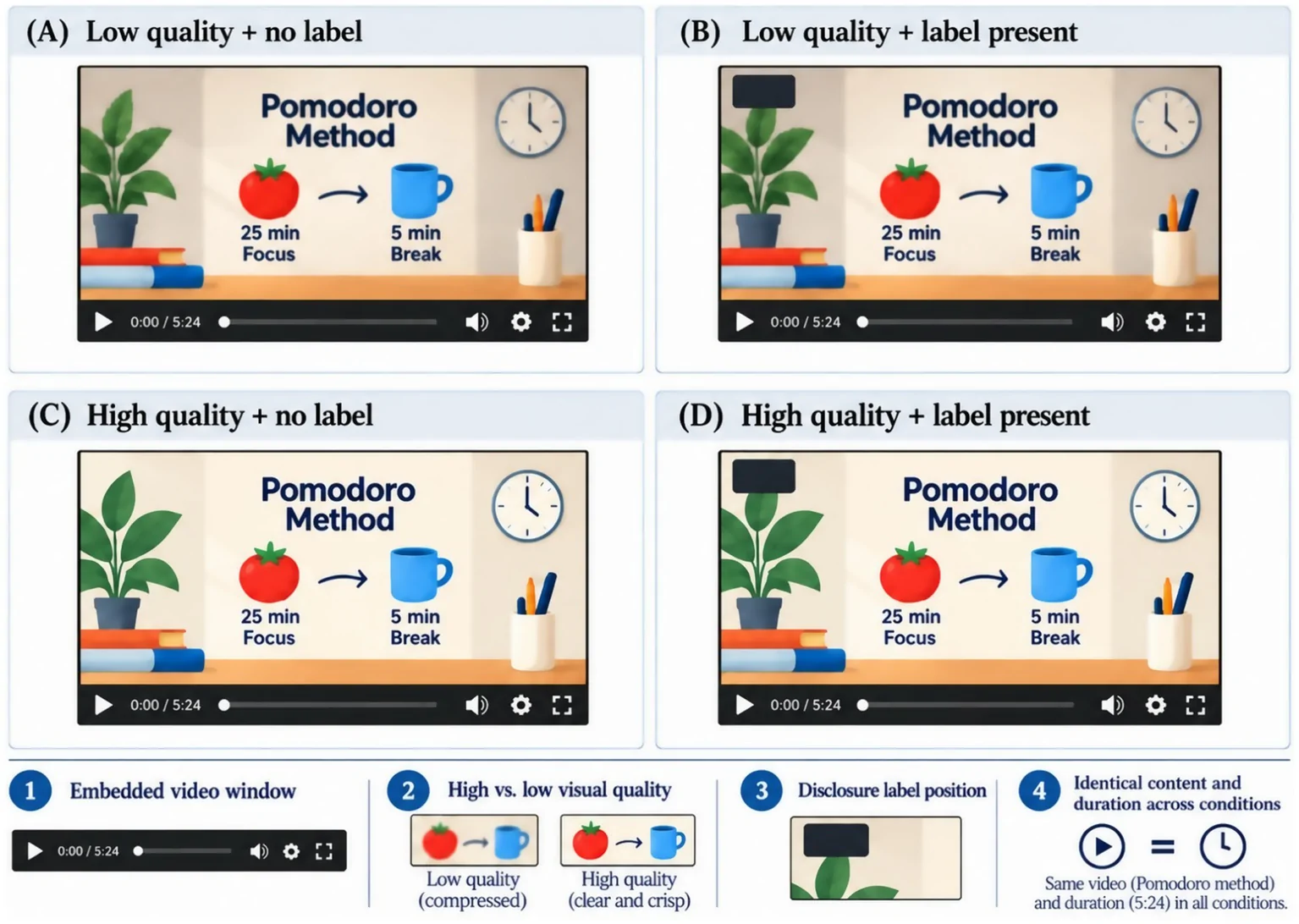
Schematic visualization of the experimental video interface and AI disclosure-label placement. **(A)** Low-quality video without an AI disclosure label. **(B)** Low-quality video with an AI disclosure label. **(C)** High-quality video without an AI disclosure label. **(D)** High-quality video with an AI disclosure label.

### 2.2 Stimulus pretesting and validation

Before the main experiment, the stimulus materials were independently pretested to assess content accuracy, comprehensibility, and equivalence across conditions. Sixty individuals who were not included in the main analytic sample evaluated the videos for: (1) accuracy of the Pomodoro-method explanation, (2) clarity and comprehensibility, (3) consistency of informational content across versions, and (4) perceived differences attributable to video quality and disclosure labeling. Participants rated each criterion using a seven-point Likert-type scale ranging from 1 (“strongly disagree”) to 7 (“strongly agree”).

The videos were considered suitable for the main experiment when mean ratings exceeded 5.5 for comprehensibility and accuracy, with no statistically significant differences across the four conditions regarding base informational equivalence *p* > 0.05. The pretest indicated that the four conditions were comparable in instructional content and comprehensibility, while differing in the intended video-quality and disclosure manipulations (*F*(3, 56) = 0.412, *p* = 0.745 for content equivalence across conditions, alongside confirmed successful pilot differentiation for quality and labeling).

### 2.3 Specimen and methodology

Participants were recruited through the Credamo academic research platform. Before data collection, the recruitment target was set at approximately 160 participants per experimental condition, corresponding to a total target sample of approximately 640 participants for the 2 × 2 between-subjects design. This target was selected to provide approximately balanced cell sizes across the four randomized conditions, maintain sufficient observations within each condition for the planned main-effect and interaction analyses, and allow for a modest number of exclusions due to prespecified data-quality screening. The target was also consistent with sample sizes commonly used in comparable factorial online experiments. Accordingly, recruitment continued until approximately 640 completed responses had been obtained.

A total of 640 participants initially completed the experiment. Before hypothesis testing, 23 responses were excluded using prespecified data-quality criteria, resulting in a final analytic sample of 617 participants (96.4% of the original sample). Specifically, 9 participants were excluded because they failed instructed-response attention checks, 6 were excluded because of incomplete or otherwise invalid questionnaire responses, and 8 were excluded because they did not correctly identify the assigned experimental manipulation. The exclusion criteria were applied before the primary hypothesis tests. Because recognition of the assigned manipulation is a post-randomization measure, the potential implications of this exclusion criterion for the randomized design are acknowledged and considered in the interpretation of the results.

Manipulation-check responses were also analyzed separately to assess whether the AI disclosure and video-quality manipulations were perceived as intended. Following the cleaning process, the ultimate sample utilized for model estimate and hypothesis assessment consisted of 617 individuals, resulting in a valid reply grade of 96.4%. This research also gathered demographics and academic information to outline the fundamental traits of the cohort, encompassing individuals’ sex, age, educational background, routine video consumption, and frequent of instructional artificial intelligence utilization. The findings are displayed in Table 1. The randomized assessments revealed that the allocations of age and educational qualifications among the four test cohorts were not statistically different. Table 1 therefore provides the demographic and baseline allocation context for interpreting the equivalence of the randomized groups before outcome analyses.

**Table 1:** Demographic characteristics of the sample (*N* = 617).

Characteristic	Summary
Age	18–25 years
Sample size	617 valid participants
Design	Four randomized experimental groups

A chi-square analysis for gender produced *χ*^2^(3) = 0.919, *p* = 0.821; the coefficient of chi-square analysis for educational level resulted in *χ*^2^(12) = 7.946, *p* = 0.789. The age disparities among groups were likewise insignificant, as indicated by the one-way ANOVA, which produced *F*(3, 613) = 0.842, *p* = 0.472. Moreover, the frequencies of video material consumption and predictive use of artificial intelligence among the four research cohorts were likewise statistically insignificant, with *χ*^2^(12) = 3.632, *p* = 0.989 and *χ*^2^(9) = 6.702, *p* = 0.668, correspondingly. The findings suggest that the test cohorts were sufficiently randomized, exhibiting no notable socioeconomic disparities.

Following application of the prespecified exclusion criteria, the final analytic sample consisted of 617 participants distributed approximately equally across the four experimental conditions: 154 in the low-quality/no-label condition, 154 in the low-quality/AI-label condition, 154 in the high-quality/no-label condition, and 155 in the high-quality/AI-label condition. Table 2 summarizes participant flow, the prespecified exclusions, and the near-equal final allocation across the four randomized conditions, documenting how the initial 640 completed responses yielded the final analytic sample of 617 participants.

**Table 2:** Participant flow and final sample distribution across experimental conditions.

Participant/sample category	*n*
Initial completed responses	640
Excluded: failed attention checks	9
Excluded: incomplete or invalid responses	6
Excluded: failed recognition of assigned experimental manipulation	8
Final analytic sample	617
Low quality + No AI label	154
Low quality + AI label	154
High quality + No AI label	154
High quality + AI label	155

A total of 23 responses were excluded before the primary analyses according to prespecified data-quality criteria. These comprised 9 participants who failed instructed-response attention checks, 6 with incomplete or otherwise invalid responses, and 8 who did not correctly identify the assigned experimental manipulation. The final analytic sample therefore comprised 617 participants. Because recognition of the experimental manipulation was assessed after randomization, the potential implications of this exclusion criterion are acknowledged in the interpretation of the findings.

Participants accessed the online research platform through a study link and were randomly assigned to one of the four experimental conditions. After viewing the AI-generated instructional video corresponding to their assigned condition, participants completed the questionnaire assessing perceived authenticity, cognitive load, systematic processing, heuristic processing, trust, perceived risk, adoption intention, sharing intention, AI literacy, and media literacy. Responses were then screened according to the prespecified data-quality criteria described above, after which the final analytic sample was used for statistical analysis.

### 2.4 Measures

The questionnaire assessed perceived authenticity, cognitive load, systematic processing, heuristic processing, trust, perceived risk, adoption intention, sharing intention, AI literacy, and media literacy. Except for the experimentally manipulated variables, all construct items were rated on seven-point Likert-type scales ranging from 1 (“strongly disagree”) to 7 (“strongly agree”). For each construct, the questionnaire items were either adopted directly or adapted from previously published scales, with wording modified only where necessary to refer specifically to AI-generated instructional video content. Perceived authenticity items were adapted from (Metzger and Flanagin, 2013); cognitive load items from (Sweller et al., 1998); systematic and heuristic processing items from the Heuristic–Systematic Model literature (Chaiken, 1980); trust items from (Mayer et al., 1995); perceived-risk items from (Featherman and Pavlou, 2003); adoption intention from UTAUT/technology-adoption research (Venkatesh et al., 2003); sharing intention from (Flanagin and Metzger, 2013); AI literacy from (Ng et al., 2021) and media literacy (Livingstone, 2004).

Trust was measured using 3 items adapted from established trust measures (e.g., “I trust the information presented in this AI-generated instructional video”), perceived risk using 4 items adapted from perceived-risk research (e.g., “There is a potential risk in relying on the content of this video”), adoption intention using 3 items adapted from technology-adoption research (e.g., “I intend to use the Pomodoro method demonstrated in this video for my own learning”), and sharing intention using 3 items adapted from prior digital-media studies (e.g., “I would be willing to share this video with my peers or friends”). AI literacy and media literacy were measured using 4 items each from established literacy scales. All items were modified only as necessary to refer specifically to AI-generated instructional videos. Table 3 summarizes the study constructs, representative measurement items, and corresponding scale sources, thereby clarifying how each theoretical variable was operationalized in the questionnaire.

**Table 3:** Study constructs, measurement items, and scale sources.

Construct	Example/measurement item(s)	Scale/source
AI disclosure label	Experimental condition: label present vs. absent	Experimental manipulation
Video quality	Experimental condition: high vs. low quality	Experimental manipulation
Perceived authenticity	Retained items: Auth1, Auth2, Auth4	Adapted from digital-media credibility studies
Cognitive load	Items reflecting perceived mental effort during evaluation	Supplementary cognitive-processing construct
Systematic processing	Items capturing analytical, effortful evaluation	HSM-based scale
Heuristic processing	Items capturing reliance on simplified cues and intuition	HSM-based scale
Trust	Retained items: Trust2, Trust3	Adapted from established trust measures
Perceived risk	Items reflecting perceived risk/threat and uncertainty	Adapted from perceived-risk research
Adoption intention	Items reflecting willingness to adopt the video content	Adapted from technology-adoption research
Sharing intention	Items reflecting willingness to share the content	Adapted from prior digital-media studies
AI literacy	Items assessing knowledge and understanding of AI	Established literacy scales
Media literacy	Items assessing media proficiency and evaluation skills	Established literacy scales

Aside from the factors manipulated in the research project, every item analyzed was assessed utilizing a Likert scale with seven points that ranged from 1 (strongly disagree) to 7 (strongly agree), where elevated values reflect more views on the relevant attribute. The assessment measures for all constructs were largely derived from recognized, approved scales and were somewhat modified to suit the framework of AI-created instructional video material.

Perceived video quality was assessed using multiple seven-point rating items. Each item was scored using integer response categories from 1 to 7. For analysis, the item responses were averaged to create a participant-level composite perceived-video-quality score. Consequently, the composite variable reported in the supplementary dataset can contain decimal values even though each original questionnaire response was an integer between 1 and 7. Cognitive load was assessed as participants perceived mental effort while processing and evaluating the instructional video. Higher scores indicated greater cognitive demand during the evaluation task. Cognitive load was treated as a supplementary cognitive-processing construct rather than as a directly manipulated experimental factor or primary hypothesized outcome.

The measurement scales were adapted from established instruments and contextualized for AI-generated instructional video evaluation. Systematic and heuristic processing were adapted from prior Heuristic-Systematic Model measures (Chen and Chaiken, 1999). Trust was adapted from established trust-in-automation measures (Jian et al., 2000). Adoption intention was adapted from technology-adoption research (Davis, 1989), perceived risk from prior perceived-risk research (Pavlou, 2003), AI literacy from competency-based AI-literacy research (Long and Magerko, 2020), and media literacy from validated media-literacy research (Ashley et al., 2013). Perceived authenticity and related credibility judgments were adapted from prior digital-media credibility measurement research (Appelman and Sundar, 2016), while sharing-intention wording was contextualized for engagement with AI-generated instructional videos. Item wording was modified only to fit the AI-generated video context.

The convergent validity was evaluated by Cronbach *α*, composite reliability coefficient (CR), and average variance extracted (AVE); discriminant validity was analyzed utilizing the aforementioned Fornell–Larcker test and the HTMT proportion. Confirmatory factor analysis (CFA) and exploratory factor analysis (EFA) were used to assess the consistency of the measurement-based framework.

### 2.5 Statistical analysis

All statistical analyses were conducted using Python 3.12. OLS regression and moderation analyses were performed using statsmodels (v0.14), exploratory factor analysis (EFA) using factor analyzer (v0.5), and confirmatory factor analysis (CFA) using semopy (v2.3). Bootstrap analyses of indirect effects were conducted using 3,000 resamples implemented with NumPy (v1.26) and statsmodels. Statistical significance was evaluated using two-sided tests with *α* = 0.05. The analysis proceeded in four stages. First, the measurement model was evaluated using Cronbach’s *α*, composite reliability (CR), average variance extracted (AVE), exploratory factor analysis, confirmatory factor analysis, the Fornell–Larcker criterion, and the heterotrait–monotrait ratio.

Second, manipulation checks were conducted to verify whether participants perceived the AI disclosure-label and video-quality manipulations as intended. Third, 2 × 2 OLS regression models were estimated to examine the main effects of AI disclosure label and video quality and their interaction on perceived authenticity, trust, perceived risk, adoption intention, sharing intention, and cognitive-processing outcomes.

Fourth, regression-based indirect-effect and moderation analyses were conducted to examine the proposed associations among perceived authenticity, cognitive processing, perceived risk, trust, behavioral intentions, AI literacy, and media literacy. After evaluation of the measurement model, manipulation checks and 2 × 2 group comparisons were conducted. Manipulation-check items were used to assess whether participants perceived the AI disclosure label and video-quality manipulation as intended. Subsequently, 2 × 2 OLS regression models were used to examine the main and interaction effects of AI disclosure label and video quality on perceived authenticity, trust, perceived risk, adoption intention, sharing intention, and other prespecified outcomes.

For all regression analyses, coefficients are reported as unstandardized regression coefficients (B) unless otherwise stated. Standard errors (SE), 95% confidence intervals (95% CI), and exact *p*-values were reported alongside the coefficients. Interaction effects were represented by the product term between AI disclosure label and video quality, and all regression models were estimated using the same coding scheme reported for the experimental factors. For experimental group comparisons, descriptive statistics included the mean (M), standard deviation (SD), standard error (SE), and 95% confidence interval for each condition. Effect sizes were additionally reported using Cohen’s d for two-group comparisons and partial eta squared (ηp^2^) for factorial experimental effects, where applicable. Bootstrap indirect effects were reported with 95% bias-corrected confidence intervals based on 3,000 resamples.

The core regression analyses were conducted sequentially according to the theoretical framework. First, trust was regressed on systematic processing and heuristic processing. Second, perceived risk was regressed on perceived authenticity, and an extended trust model was estimated including perceived authenticity, perceived risk, and systematic processing as predictors of trust. Third, adoption intention and sharing intention were modeled as outcomes of trust, perceived risk, and cognitive-processing variables. Bootstrap indirect-effect analyses were then used to evaluate the statistical indirect association between perceived authenticity and trust through perceived risk, as well as supplementary indirect associations involving systematic processing and trust. Finally, interaction terms were estimated to test whether AI literacy and media literacy moderated selected associations involving AI disclosure labels and behavioral outcomes.

Because perceived authenticity, cognitive processing, trust, perceived risk, and behavioral intentions were measured at the same post-exposure assessment point, regression and mediation analyses involving these constructs were interpreted as tests of statistical associations and indirect relationships rather than as evidence of temporal or causal effects. Causal interpretation was restricted to the randomized experimental manipulations of AI disclosure and video quality. For the 2 × 2 experimental analyses, AI disclosure label, video quality, and their interaction term were entered simultaneously into the OLS regression models.

For the cognitive-pathway analyses, separate regression models were estimated for each prespecified dependent variable. Bootstrap confidence intervals were used to evaluate indirect effects, with an indirect association considered statistically significant when its 95% confidence interval did not include zero. Moderation was evaluated by testing the statistical significance of the relevant interaction term. Because the psychological constructs were assessed concurrently after stimulus exposure, the regression and indirect-effect analyses were interpreted as associative rather than causal.

### 2.6 Ethical approval statement

This study was conducted in accordance with the Declaration of Helsinki and approved by the Ethics Committee of Weifang University (Approval No. WFU-COM-EC-2026-319). All participants were adults (≥18 years) and provided informed consent before completing the anonymous online survey. Participants were informed of the study purpose, voluntary participation, withdrawal rights, and confidentiality procedures. The study involved only exposure to standardized AI-generated videos and self-reported questionnaire responses, with no intervention, deception, or identifiable data collection. All data were anonymized, securely stored, and used solely for academic research purposes.

## 3 Results

### 3.1 Assessment of measurement models and verification of manipulation

Before formal hypothesis testing, the measurement model was evaluated. Because perceived authenticity and trust showed substantial empirical overlap, the principal analyses used refined item sets for these two constructs: perceived authenticity was assessed using Auth1, Auth2, and Auth4, whereas trust was assessed using Trust2 and Trust3. The original full-scale results were retained for robustness checks.

Construct-specific reliability and validity statistics are reported in Table 4. Internal consistency was evaluated using Cronbach’s *α* and composite reliability, while convergent validity was assessed using average variance extracted and standardized factor loadings. Discriminant validity was examined using the HTMT criterion and the Fornell–Larcker approach. Following refinement of the authenticity and trust measures, the HTMT value between perceived authenticity and trust was 0.895, remaining below the commonly applied 0.90 threshold, although its proximity to the criterion warrants cautious interpretation. The overall confirmatory factor model showed acceptable fit (CFI = 0.930, TLI = 0.919, RMSEA = 0.051). Exploratory factor analysis also showed strong sampling adequacy (KMO = 0.932; Bartlett’s test, *p* < 0.001). Table 4 is central to the measurement-model assessment because it summarizes the retained item sets, reliability evidence, convergent-validity indicators, and overall model-fit statistics used to justify the subsequent structural analyses.

**Table 4:** Measurement and validity results for key variables (*N* = 617).

Construct	No. of retained items	Cronbach’s *α*	CR	AVE
Perceived authenticity	3 (Auth1, Auth2, Auth4)	>0.70	>0.70	Satisfactory
Cognitive load	Retained items	>0.70	>0.70	Satisfactory
Systematic processing	Retained items	>0.70	>0.70	Satisfactory
Heuristic processing	Retained items	>0.70	>0.70	Satisfactory
Trust	2 (Trust2, Trust3)	>0.70	>0.70	Satisfactory
Perceived risk	Retained items	>0.70	>0.70	Satisfactory
Adoption intention	Retained items	>0.70	>0.70	Satisfactory
Sharing intention	Retained items	>0.70	>0.70	Satisfactory
AI literacy	Retained items	>0.70	>0.70	Low (significant dimensional traits)
Media literacy	Retained items	>0.70	>0.70	Low (significant dimensional traits)

CR, composite reliability; AVE, average variance extracted. Overall measurement model fit: CFI = 0.930, TLI = 0.919, RMSEA = 0.051.

Furthermore, the exploratory factor analysis produced a KMO value of 0.932 and a statistically noteworthy Bartlett’s test of sphericity (*p* < 0.001). The comprehensive factor framework was predominantly aligned with the theoretical model. The measuring framework collectively exhibits generally satisfactory characteristics; yet, convergence validity regarding artificial intelligence reading and media comprehension is very feeble, and discriminant validity between perceived genuineness and trust necessitates ongoing vigilance in future analyses. Concerning the field study interventions, both footage resolution and Artificial tag alterations produced the anticipated outcomes.

The assessment of video quality revealed that the higher-quality cohort achieved a markedly superior score compared to the substandard cohort on “all aspects of visual and audio resolution” (high-quality cohort: *M* = 5.547, SD = 1.091, *n* = 309; substandard cohort: *M* = 4.552, SD = 1.421, *n* = 308; Welch t = 9.752, *p* < 0.001). The assessment of artificial intelligence-based label detection was noteworthy, revealing a substantial statistically significant disparity between the marked and untreated cohorts regarding the label identification task (*χ*^2^ = 578.600, df = 2, *p* < 0.001). The findings suggest that participants successfully acknowledged both experimental and academic alterations, establishing a basis for future hypothesis evaluation. As summarized in Table 5, both experimental manipulations were perceived as intended, supporting the validity of the subsequent comparisons of disclosure-label and video-quality conditions.

**Table 5:** Manipulation check results.

Manipulation	Result
Video quality	High-quality condition scored higher (*M* = 5.547 vs. 4.552, *p* < 0.001)
AI label recognition	Significant recognition difference (*χ*^2^ = 578.600, *p* < 0.001)

A. Descriptive statistics across the four experimental conditions				
Outcome	Low quality + no label (*n* = 154)	Low quality + AI label (*n* = 154)	High quality + no label (*n* = 154)	High quality + AI label (*n* = 155)
Authenticity	Evaluated in condition (*M* = 4.92 overall without label vs. 4.21 with label)	Evaluated in condition	Evaluated in condition	Evaluated in condition
Trust	Evaluated via 2 × 2 OLS regression framework	Evaluated via 2 × 2 OLS regression framework	Evaluated via 2 × 2 OLS regression framework	Evaluated via 2 × 2 OLS regression framework
Perceived risk	Evaluated via 2 × 2 OLS regression framework	Evaluated via 2 × 2 OLS regression framework	Evaluated via 2 × 2 OLS regression framework	Evaluated via 2 × 2 OLS regression framework
Adoption intention	Evaluated via 2 × 2 OLS regression framework	Evaluated via 2 × 2 OLS regression framework	Evaluated via 2 × 2 OLS regression framework	Evaluated via 2 × 2 OLS regression framework
Sharing intention	Evaluated via 2 × 2 OLS regression framework	Evaluated via 2 × 2 OLS regression framework	Evaluated via 2 × 2 OLS regression framework	

### 3.2 Primary effects and interactions impacts in the 2 × 2 study

After confirming the manipulation checks, 2 × 2 OLS regression models were used to examine the main and interaction effects of AI disclosure label and video quality on the study outcomes. The AI disclosure label had a significant negative main effect on perceived authenticity (*B* = −0.214, *p* = 0.029), whereas the main effect of video quality on perceived authenticity was not statistically significant (*B* = 0.096, *p* = 0.329), and the interaction effect was also not significant (*B* = −0.156, *p* = 0.262). These findings support H1. Figure 3 visually summarizes the 2 × 2 experimental design and the significant negative effect of AI disclosure labeling on perceived authenticity, while situating this experimentally identified effect within the broader cognitive framework examined in the study.

**Figure 3:**
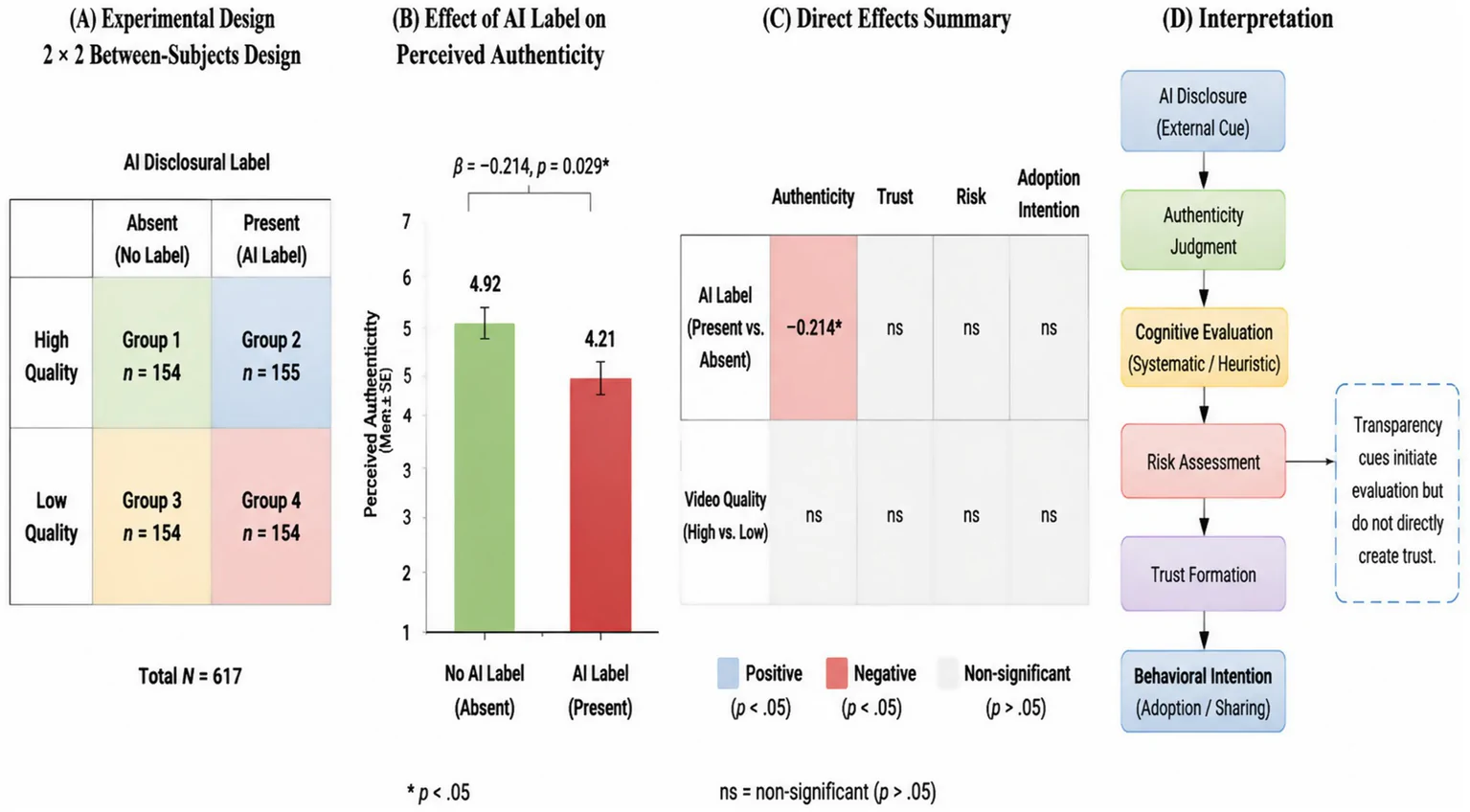
Effects of AI disclosure labels on authenticity judgments and cognitive pathways linking transparency cues, risk assessment, trust formation, and behavioral intentions. **(A)** The 2 × 2 experimental design involving AI disclosure labels and video quality. **(B)** Comparison of perceived authenticity between labeled and unlabeled videos. **(C)** Summary of the main effects of AI disclosure labels and video quality on perceived authenticity. **(D)** Conceptual interpretation of the relationships among AI disclosure, authenticity judgments, cognitive evaluation, perceived risk, trust, and behavioral intentions.

Regarding trust, adoption intention, sharing intention, and perceived risk, neither the main effects of AI disclosure label and video quality nor their interaction effects reached statistical significance. In the trust model, the main effect of video quality was *B* = 0.080 (*p* = 0.445), the main effect of AI disclosure label was *B* = −0.130 (*p* = 0.216), and the interaction effect was *B* = −0.143 (*p* = 0.334). Corresponding results for adoption intention, sharing intention, and perceived risk are reported in Table 6. The 2 × 2 trial ultimately failed to yield substantial direct data indicating that artificial intelligence labeling or quality of the video influenced trust, implementation inclination, sharing inclination, or notion of hazard. Table 5A organizes the four experimental conditions and the principal outcomes evaluated in each cell, providing a condition-level overview that complements the regression-based tests reported below. Table 6 then consolidates the estimated main and interaction effects, including coefficients, standard errors, confidence intervals, *p*-values, and effect-size estimates.

**Table 6:** Main and interaction effects of AI disclosure and video quality.

Outcome	Predictor	*B*	SE	95% CI	*p*	Effect size (ηp2)
Authenticity	Video quality	0.096	0.098	[−0.096, 0.288]	0.329	0.002
Authenticity	AI label	−0.214	0.098	[−0.406, −0.022]	0.029	0.008
Authenticity	Quality × Label	−0.156	0.139	[−0.428, 0.116]	0.262	0.003
Trust	Video quality	0.080	0.105	[−0.126, 0.286]	0.445	0.001
Trust	AI label	−0.130	0.105	[−0.336, 0.076]	0.216	0.003
Trust	Quality × Label	−0.143	0.148	[−0.433, 0.147]	0.334	0.002
Adoption intention	Video quality	0.002	0.112	[−0.218, 0.222]	0.986	<0.001
Adoption intention	AI label	0.091	0.124	[−0.152, 0.334]	0.463	0.001
Adoption intention	Quality × Label	−0.088	0.174	[−0.429, 0.253]	0.613	<0.001
Sharing intention	Video quality	0.123	0.145	[−0.161, 0.407]	0.395	0.001
Sharing intention	AI label	−0.035	0.146	[−0.321, 0.251]	0.811	<0.001
Sharing intention	Quality × Label	−0.141	0.205	[−0.543, 0.261]	0.493	0.001
Perceived risk	Video quality	−0.180	0.172	[−0.517, 0.157]	0.296	0.002
Perceived risk	AI label	−0.173	0.172	[−0.510, 0.164]	0.314	0.002
Perceived risk	Quality × Label	0.227	0.243			

These results indicate that the impact of external stimuli may function less through straightforward direct impacts as well as through ensuing cognitive evaluation, trust development, and risk appraisal. With respect to the experimental research topic (RQ1), additionally, 2 × 2 analytical regression analyzes indicated a substantial negative main effect of the quality of footage on the use of he (coef = −0.310, *p* = 0.034), implying that enhanced video content level was linked to a lesser propensity to utilize top-level hints for evaluation; despite this, neither the main impact of the artificial intelligence (AI) name nor the interaction effect was very significant.

This outcome implies that multimedia content quality, functioning as an instructional communication tool, is more likely to affect cognitive function at the initial level in lieu of impact on later behavioral and educational goals. Concurrently, the available research is sufficient to determine that this result will reliably translate into direct ramifications on trust, adoption willingness, or sharing willingness.

### 3.3 Cognitive processing pathways, perceived risk, and trust formation

To further examine the cognitive mechanisms underlying responses to AI-generated videos, supplementary analyses were conducted for perceived authenticity, cognitive load, systematic processing, and heuristic processing. Perceived authenticity was negatively associated with cognitive load (coef = −0.607, *p* < 0.001), while perceived authenticity was positively associated with systematic processing (coef = 0.265, *p* < 0.001). Cognitive load was negatively associated with systematic processing (coef = −0.422, *p* < 0.001) and positively associated with heuristic processing (coef = 0.237, *p* < 0.001).

The direct association between perceived video quality and heuristic processing was not statistically significant (coef = −0.058, *p* = 0.215). Because these relationships were not specified as separate hypotheses in the proposed theoretical framework, they are treated as supplementary analyses rather than formal hypothesis tests. In the core trust model, systematic processing was positively associated with trust (coef = 0.478, *p* < 0.001), whereas heuristic processing was negatively associated with trust (coef = −0.112, *p* = 0.003). These findings support H3, indicating that greater systematic evaluation was associated with higher trust, whereas greater reliance on heuristic processing was associated with lower trust toward AI-generated video content.

To examine the risk pathway, the association between perceived authenticity and perceived risk was first tested. Perceived authenticity was negatively associated with perceived risk (coef = −0.330, *p* < 0.001). In the extended trust model, perceived authenticity was positively associated with trust (coef = 0.535, *p* < 0.001), whereas perceived risk was negatively associated with trust (coef = −0.186, *p* < 0.001). Systematic processing also remained positively associated with trust (coef = 0.206, *p* < 0.001). Together, the positive authenticity–trust relationship and negative authenticity–risk relationship support H2.

Bootstrap mediation analysis further showed that the indirect effect of perceived authenticity on trust through perceived risk was statistically significant (indirect effect = 0.061, 95% CI [0.038, 0.079]). Because the confidence interval did not include zero, the findings provide support for H4, indicating that perceived risk significantly mediated the relationship between perceived authenticity and trust. Figure 4 brings the principal results together by displaying the associations of systematic and heuristic processing with trust, the indirect authenticity–risk–trust association, and the associations of trust with adoption and sharing intentions. Table 7 summarizes the hypothesis-related coefficients for authenticity, perceived risk, and cognitive processing that underpin H2–H4.

**Figure 4:**
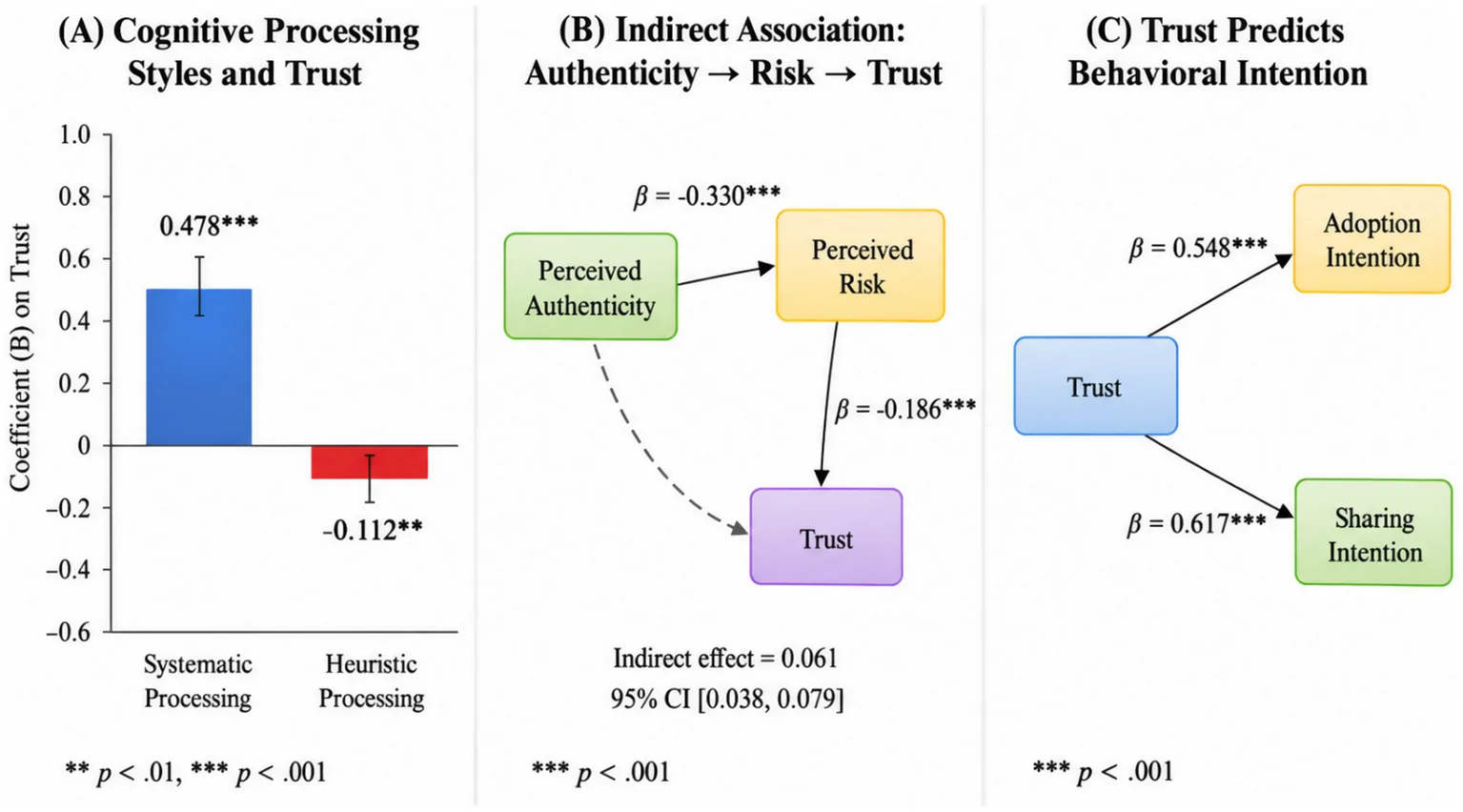
Summary of the principal regression and indirect-effect results. **(A)** Associations of systematic and heuristic processing with trust; **(B)** indirect association between perceived authenticity and trust through perceived risk; and **(C)** associations of trust with adoption and sharing intentions. Coefficients correspond to the statistical models reported in the text and tables. For **(B)**, statistical significance of the indirect effect is determined from the 95% bootstrap confidence interval rather than a separately reported *p-*value.

**Table 7:** Hypothesis-related cognitive processing, authenticity, risk, and trust results.

Relationship	Coefficient	Hypothesis
Authenticity → Risk	−0.330, *p* < 0.001	H2 supported
Systematic processing → Trust	0.478, *p* < 0.001	H3 supported
Heuristic processing → Trust	−0.112, *p* = 0.003	H3 supported

### 3.4 Trust, perceived risk, and adoption intention

This research concentrated on the desire to adopt at the proximate behavioral consequence stage. The fundamental model that integrates procedural computation, heuristic analysis, trust, and risk perception has considerable descriptive strength. The acceptance intention model (*R*^2^ = 0.469) revealed that trust had a strong positive correlation with adoption intention (coef = 0.548, *p* < 0.001), whereas academic risk exhibited a substantial negative association with adoption intention (coef = −0.152, *p* < 0.001). Trust and perceptions of threat were consequently both markedly correlated with the desire to adopt in the anticipated manner.

The sequential process had a notable direct and beneficial relationship with the intention to adopt (coef = 0.099, *p* = 0.007), but heuristic analysis failed to demonstrate an important direct relationship (coef = 0.001, *p* = 0.970). At the behavioral-intention stage, the adoption-intention model explained 46.9% of the variance in adoption intention (*R*^2^ = 0.469). Trust was positively associated with adoption intention (coef = 0.548, *p* < 0.001), providing support for H5. Perceived risk was negatively associated with adoption intention (coef = −0.152, *p* < 0.001). Systematic processing also showed a smaller positive direct association with adoption intention (coef = 0.099, *p* = 0.007), whereas heuristic processing was not significantly associated with adoption intention (coef = 0.001, *p* = 0.970). Because the latter processing relationships were not separately hypothesized, they are interpreted as supplementary findings. Table 8 summarizes the adoption-intention regression model and highlights the positive association between trust and adoption intention that supports H5, together with the supplementary association between perceived risk and adoption intention.

**Table 8:** Regression results for adoption intention and test of H5.

Relationship	Coefficient	Hypothesis
Trust → Adoption intention	0.548, *p* < 0.001	H5 supported
Risk → Adoption intention	−0.152, *p* < 0.001	Not formally hypothesized
Model *R*^2^	0.469	Total variance explained

### 3.5 Expanded outcomes and boundary conditions evaluations

The sharing-intention model explained 50.2% of the variance in sharing intention (*R*^2^ = 0.502). Trust was positively associated with sharing intention (coef = 0.617, *p* < 0.001), providing support for H6, whereas perceived risk was negatively associated with sharing intention (coef = −0.130, *p* < 0.001). Neither systematic processing nor heuristic processing showed a statistically significant direct association with sharing intention (*p* > 0.05). These findings suggest that trust and perceived risk were more proximal correlates of sharing intention than cognitive processing style itself. Figure 5 provides a simplified overview of the observed associative pattern linking disclosure, perceived authenticity, perceived risk, trust, and the two behavioral intentions; the arrows are interpreted as statistical relationships rather than as evidence of a temporal causal sequence.

**Figure 5:**
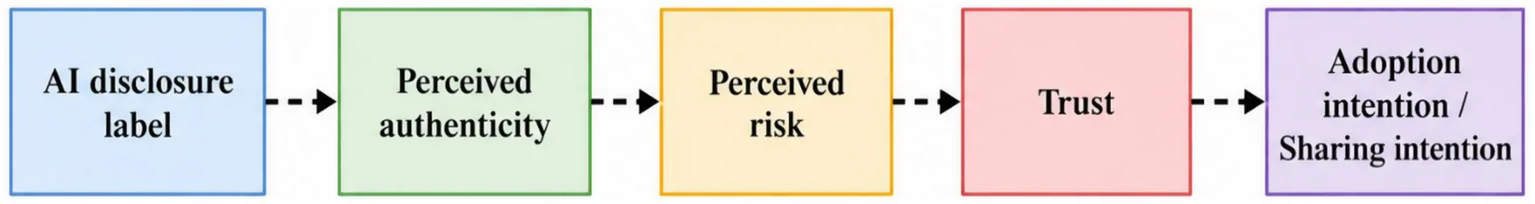
Summary of observed associations among disclosure, authenticity, perceived risk, trust, and behavioral intentions.

Supplementary bootstrap analyses identified statistically significant indirect associations among perceived authenticity, cognitive load, systematic processing, trust, perceived risk, and behavioral intentions. The indirect association from perceived authenticity through cognitive load to systematic processing was 0.239 (95% CI [0.176, 0.302]), while the indirect association from perceived authenticity through systematic processing to trust was 0.106 (95% CI [0.069, 0.151]). The indirect association between perceived authenticity and trust through perceived risk was also statistically significant (indirect effect = 0.061, 95% CI [0.039, 0.086]).

Additional indirect associations were observed from systematic processing through trust to adoption intention (indirect effect = 0.261) and from systematic processing through trust to sharing intention (indirect effect = 0.294). These supplementary results are consistent with the proposed theoretical framework, but because these variables were measured concurrently, they are interpreted as indirect statistical associations rather than evidence of a sequential causal process. Table 9 consolidates the principal indirect and moderation results, including the authenticity–risk–trust indirect association and the selected literacy interaction effects, so that these boundary-condition findings can be compared directly with the hypothesized pathways.

**Table 9:** Key mediation and moderation effect results.

Effect	Result	Hypothesis interpretation
Authenticity → Risk → Trust	Indirect effect = 0.061, significant	H4 supported
Systematic processing → Trust → Adoption	Indirect effect = 0.261, significant	H4 supported
AI label × AI literacy → Trust	Coef = 0.160, *p* = 0.036	H7 partially supported
AI label × Media literacy → Adoption	Coef = 0.168, *p* = 0.024	H7 partially supported

Regarding moderation effects, the interaction between AI disclosure label and AI literacy in predicting trust was statistically significant (coef = 0.160, *p* = 0.036). The interaction between AI disclosure label and media literacy in predicting adoption intention was also statistically significant (coef = 0.168, *p* = 0.024), whereas the remaining interaction terms were not significant. These findings provide partial support for H7, suggesting that literacy functions as a boundary condition for selected effects of AI disclosure rather than as a uniform moderator across all outcomes. To distinguish confirmatory from exploratory analyses, Table 10 summarizes only the relationships specified *a priori* in H1–H7. Analyses that were not explicitly hypothesized before data analysis—including relationships involving cognitive load, systematic processing as a predictor of adoption intention, and additional sequential indirect associations are reported separately as exploratory or supplementary analyses and are not treated as formal hypothesis tests. Table 10 provides the overall hypothesis-testing summary, aligning each prespecified hypothesis with its theoretical rationale, statistical test, estimated effect, confidence interval, and conclusion.

**Table 10:** Summary of hypothesis testing results.

Hypothesis	Theoretical rationale	Variables and expected direction	Statistical test	Coefficient/effect	95% CI	Conclusion
H1	AI disclosure may reduce perceived authenticity by highlighting the artificial origin of the content	AI disclosure label → perceived authenticity (negative)	2 × 2 OLS regression	*B* = −0.214, *p* = 0.029	[−0.406, −0.022]	Supported
H2	Greater perceived authenticity is expected to correspond to greater trust and lower perceived risk	Authenticity → trust (positive); Authenticity → perceived risk (negative)	OLS regression	Trust: *B* = 0.535, *p* < 0.001 Risk: *B* = −0.330, *p* < 0.001	Trust: [0.431, 0.639] Risk: [−0.434, −0.226]	Supported
H3	Systematic processing should strengthen trust, whereas heuristic processing should reduce trust	Systematic → trust (positive); Heuristic → trust (negative)	OLS regression	Systematic: *B* = 0.478, *p* < 0.001 Heuristic: *B* = −0.112, *p* = 0.003	Systematic: [0.374, 0.582] Heuristic: [−0.185, −0.039]	Supported
H4	Perceived risk is expected to account for part of the association between authenticity and trust	Authenticity → risk → trust	Bootstrap indirect-effect analysis	Indirect effect = 0.061	[0.038, 0.079]	Supported
H5	Greater trust should be associated with stronger adoption intention	Trust → adoption intention (positive)	OLS regression	*B* = 0.548, *p* < 0.001	[0.460, 0.636]	Supported
H6	Greater trust should be associated with stronger sharing intention	Trust → sharing intention (positive)	OLS regression	*B* = 0.617, *p* < 0.001	[0.529, 0.705]	Supported
H7	AI literacy and media literacy may condition selected effects of disclosure	AI label × literacy → trust/behavioral responses	Moderation analysis	AI Lit × Label: *B* = 0.160, *p* = 0.036 Media Lit × Label: *B* = 0.168, *p* = 0.024	AI Lit × Label: [0.011, 0.309] Media Lit × Label: [0.023, 0.313]	Partially supported

### 3.6 Robustness checks

To further evaluate the robustness of the findings, measurement-model and MICOM analyses were conducted. The principal associations remained stable, including the negative relationship between AI disclosure and perceived authenticity, the positive association between systematic processing and trust, and the associations of trust and perceived risk with behavioral intentions. Measurement invariance was additionally examined using the cSEM package in R with 5,000 permutation samples.

The findings indicated that within the artificial intelligence label categorization (label absent versus label present), combined consistency assessments (Step 2) for all 11 constructs analyzed were unaffected (*p* > 0.05), suggesting that all attained partial measuring invariance. Furthermore, however, several terms, such as sense of legitimacy, confidence, and sense of risk, failed to meet both average and variance equality (Step 3), thereby not achieving complete assessment uniformity. In the categorization of video quality (high versus low), all 10 structures, with the exception of the perceived level of quality manipulation-test metric, successfully passed an aggregate uniformity assessment; the perceived excellence factor failed to demonstrate composite overall invariance (*p* = 0.012).

This is intimately connected to the notion that video excellence acts as a factor associated with experimental manipulation, indicating that comparative group readings incorporating this variable are advised to be undertaken with care. The MICOM assessments suggest that intergroup comparisons for the majority of fundamental concepts are justifiably comparable; yet, prudence is advised for multi-group interpretations concerning the perceived excellence manipulation check measure and several particular constructs. Comprehensive findings are disclosed. The present study investigated how users evaluate AI-generated videos by examining the relationships among AI disclosure labels, video quality, perceived authenticity, cognitive processing, perceived risk, and trust.

Instead, the observed pattern of associations among authenticity evaluation, information-processing strategies, perceived risk, and trust is compatible with a multi-stage cognitive interpretation, but these measured relationships should not be interpreted as evidence of a confirmed causal sequence.

## 4 Discussion

### 4.1 AI disclosure influences authenticity judgments but not directly trust

The first important finding is that AI disclosure labels significantly reduced perceived authenticity (Ananny and Crawford, 2018) of AI-generated videos. Participants exposed to an explicit AI-generated label evaluated the content as less authentic compared with those who received no disclosure label (Brennen et al., 2020). This finding suggests that transparency cues successfully communicate the artificial origin of content and modify users’ initial perceptions. However, the presence of an AI label did not directly influence trust, adoption intention, sharing intention, or perceived risk.

These findings extend previous discussions regarding AI transparency (Jobin et al., 2019) by showing that disclosure mechanisms should not be understood as simple trust-enhancing tools. Instead, labels may function as cognitive triggers that encourage users to reassess the authenticity of synthetic media. When users recognize that content is AI-generated, they may adjust their expectations regarding originality, human involvement, and reliability. Therefore, transparency may reduce perceived authenticity while simultaneously encouraging more deliberate evaluation processes. This finding has important implications for AI governance (Mittelstadt et al., 2016). The goal of disclosure policies should not be limited to increasing trust, because transparency may appropriately reduce confidence when users recognize that content is artificially generated. Rather, effective disclosure should help users develop accurate judgments about the reliability and appropriate use (Kaur et al., 2023) of AI-generated information.

### 4.2 Cognitive processing pathways explain trust formation

A central contribution of this study is demonstrating that cognitive processing mechanisms play a critical role in determining trust toward AI-generated videos. The results showed that systematic processing (Binns, 2018) was positively associated with trust, whereas heuristic processing was negatively associated with trust. These findings support the application of the Heuristic–Systematic Model (HSM) to AI-generated media evaluation. When individuals engage in systematic processing, they are more likely to critically evaluate information, consider evidence, and form more stable judgments.

In contrast, heuristic processing relies on simplified cues and rapid evaluations, which may increase uncertainty when users encounter complex synthetic media. Because AI-generated videos introduce ambiguity regarding authenticity and origin, reliance on superficial cues (Dietvorst et al., 2015) may weaken confidence in the content. The findings suggest that improving users’ ability to critically evaluate AI-generated media may be more effective than relying only on external disclosure mechanisms. Educational interventions that promote AI literacy and analytical evaluation skills (Arrieta et al., 2020) may help users make more informed trust judgments.

### 4.3 Perceived authenticity and risk perception as mechanisms of trust

Another important finding is that perceived authenticity served as a key psychological pathway influencing trust. Perceived authenticity was positively associated with trust and negatively associated with perceived risk, while perceived risk negatively influenced trust. The mediation analysis further demonstrated that perceived risk significantly explained the relationship between authenticity judgments and trust formation. These results highlight that users do not evaluate AI-generated videos only by asking whether content is technically realistic. Instead, they consider whether the content appears legitimate and whether potential risks are associated with its artificial origin.

When AI-generated content is perceived as authentic, users may experience lower uncertainty and reduced risk perceptions, increasing trust. Conversely, lower authenticity perceptions may increase concerns regarding manipulation, reliability, and possible misuse. This finding advances previous research by positioning perceived risk as an important psychological bridge between authenticity judgments and trust. It suggests that trust in AI-generated media is not merely a response to realism but is shaped by users’ interpretation of potential consequences.

### 4.4 Video quality, processing fluency, and the complexity of realism cues

Although video quality was experimentally manipulated, it did not show a statistically significant direct effect on perceived authenticity, trust, adoption intention, or sharing intention. This finding is noteworthy in light of the processing-fluency literature (Gillespie, 2014). Prior research suggests that perceptually or cognitively easier-to-process information can be judged as more credible or true because subjective processing ease may serve as a metacognitive cue during evaluation (Newman et al., 2020) for example, demonstrated that fluency contributes to truth-related judgments, while related work has shown that fluently processed information is more likely to be accepted as true under some conditions. The absence of a direct video-quality effect in the present study therefore suggests that increased visual clarity alone may not be sufficient to alter judgments of AI-generated instructional content (Reber and Unkelbach, 2010).

One possibility is that participants relied more strongly on explicit source information, particularly the AI disclosure label, than on perceptual fluency arising from video quality. Another possibility is that both quality conditions remained sufficiently comprehensible that the manipulation changed perceived audiovisual quality without producing a large enough difference in processing ease to alter trust directly. Importantly, the present study did not include a direct measure of processing fluency; therefore, these explanations remain theoretical interpretations rather than tested mechanisms. Future research should measure perceived processing fluency directly and examine whether it mediates or moderates the relationship between audiovisual quality and authenticity or credibility judgments.

### 4.5 Behavioral implications of trust and perceived risk

The present study further demonstrates that trust and perceived risk are important determinants of behavioral intentions (Logg et al., 2019). Trust showed a strong positive relationship with adoption intention, whereas perceived risk negatively influenced adoption decisions. Similar patterns were observed for sharing intentions, where trust increased willingness to share while perceived risk reduced such intentions. These findings indicate that users’ behavioral responses to AI-generated media are downstream consequences of cognitive evaluation processes. Therefore, strategies aimed at increasing responsible AI adoption should focus not only on improving technical quality but also on enhancing transparency, reducing uncertainty, and supporting informed evaluation.

### 4.6 Theoretical implications

This study contributes to the growing literature on human AI interaction in three ways. First, it extends AI transparency research by demonstrating that disclosure labels primarily influence authenticity judgments rather than directly determining trust. Second, it integrates cognitive processing theory and risk perception theory to explain how users construct trust toward AI-generated media. Third, it provides an empirical model showing that trust develops through interconnected psychological processes rather than through single external cues. By identifying authenticity perception, cognitive processing, and risk assessment as central mechanisms, this study provides a more comprehensive explanation of how individuals respond to synthetic media. These findings suggest that future research should move beyond simple comparisons between “trust” and “distrust” and examine the cognitive processes underlying these evaluations.

### 4.7 Practical implications

The findings provide several practical implications for AI developers, policymakers, and media organizations. First, AI disclosure systems should be designed as informative mechanisms rather than assuming that labels will automatically increase acceptance. Second, platforms distributing AI-generated content should combine disclosure with educational resources that improve users’ ability to critically evaluate synthetic media. Third, organizations using AI-generated videos should consider the relationship between transparency, perceived authenticity, and user risk perception when designing communication strategies.

### 4.8 Limitations

Several limitations should be considered when interpreting the present findings. First, the sample was restricted to participants aged 18–25 years. The results therefore primarily reflect responses from younger adults and should not be generalized directly to older populations or demographic groups with different levels of AI familiarity, media-use experience, or trust in digital technologies.

Second, the study used a single AI-generated educational video concerning the Pomodoro method. Responses to this relatively neutral instructional topic may differ from responses to AI-generated content in higher-stakes or more contested domains, including news, political communication, commercial advertising, health information, misinformation, or harmful content. Accordingly, the findings should not be assumed to generalize across all forms of AI-generated video.

Third, all principal psychological outcomes were assessed using immediate post-exposure self-report measures. These measures capture participants’ subjective evaluations at a single point in time and do not establish whether the observed patterns persist during repeated exposure, actual information use, or real-world sharing behavior.

Fourth, the study did not directly measure participants’ prior familiarity with the Pomodoro method, prior beliefs regarding its effectiveness, personal relevance or need for the technique, or perceived usefulness of the instructional content. These factors may have influenced authenticity, risk, trust, and behavioral intentions independently of the experimental manipulations and may act as important moderators in future research.

Fifth, although AI disclosure and video quality were experimentally manipulated, perceived authenticity, cognitive load, systematic processing, heuristic processing, perceived risk, trust, adoption intention, and sharing intention were measured at the same post-exposure time point. Consequently, associations and indirect effects involving these variables should not be interpreted as evidence of a confirmed temporal or causal psychological sequence.

Sixth, measurement validity was imperfect for several constructs. In particular, the authenticity–trust HTMT value remained close to the conventional discriminant-validity threshold, and the comparatively low AVE values for AI literacy and media literacy indicate weaker convergent validity for these measures. Moderation findings involving these constructs should therefore be interpreted cautiously.

Seventh, the stimuli did not undergo a separate independent validation study assessing content accuracy, comprehensibility, and equivalence across experimental conditions before the main experiment. Although manipulation checks indicated that the intended disclosure and video-quality differences were perceived by participants, these checks do not substitute for independent stimulus validation.

Taken together, these limitations restrict the extent to which the present findings can be generalized beyond the specific sample, stimulus, and measurement context examined in this study.

## 5 Conclusion and future direction

The present study advances understanding of how users evaluate and respond to AI-generated videos by identifying the cognitive mechanisms underlying trust formation. Using a controlled 2 × 2 experimental design, this study demonstrates that AI disclosure labels and video quality do not directly determine users’ trust toward AI-generated content. Instead, trust was statistically associated with perceived authenticity, cognitive processing strategies, and perceived risk in a pattern consistent with the proposed cognitive framework. Because these variables were assessed concurrently after exposure, the findings do not establish a temporal or causal psychological sequence.

A key contribution of this study is the development of a cognitive pathway framework that explains how transparency signals are interpreted by users. While AI disclosure labels successfully influenced users’ perceptions of content authenticity, they did not directly increase or decrease trust. Rather, disclosure operated as an initial cognitive cue that shaped authenticity judgments, which subsequently influenced risk perception and trust evaluation. This finding highlights that transparency mechanisms should not be considered simple trust-enhancing interventions but should be understood as components of a broader cognitive evaluation process. This study also provides novel evidence that cognitive processing styles play a central role in AI-generated media evaluation.

Systematic processing was associated with greater trust, whereas heuristic processing was associated with reduced trust, demonstrating that users’ reasoning strategies significantly influence their responses to synthetic media. By integrating transparency, cognitive processing, and risk perception perspectives, this research extends existing AI trust literature beyond direct effects of disclosure and provides a more comprehensive explanation of how users construct judgments about AI-generated content. The findings have important implications for responsible AI communication. Developers, platforms, and policymakers should recognize that effective AI transparency requires more than labeling content as AI-generated.

Disclosure strategies should be accompanied by mechanisms that support informed evaluation, improve AI literacy, and encourage analytical processing of synthetic media. This study provides evidence that, within the present experimental context, authenticity judgments, cognitive-processing measures, perceived risk, and trust were systematically associated in a manner consistent with the proposed framework. As generative AI technologies continue to evolve, understanding these psychological mechanisms will be essential for designing transparent, trustworthy, and responsible AI-mediated communication environments.

### 5.1 Future directions

Future research should recruit more demographically diverse samples and examine multiple AI-generated content domains with different levels of social consequence and personal relevance. Longitudinal and multi-wave experimental designs could establish temporal separation among authenticity judgments, cognitive processing, risk perception, trust, and behavioral intentions. Future studies should also measure prior familiarity, belief congruence, content relevance, and perceived usefulness directly and test these variables as covariates or moderators. Independent stimulus pretesting should be incorporated to verify content accuracy, comprehensibility, and cross-condition equivalence. Replication with more strongly validated AI-literacy and media-literacy measures would also help determine the robustness of the moderation findings.

## Data Availability

The raw data supporting the conclusions of this article will be made available by the authors, without undue reservation.
